# Self-diffusion in garnet-type Li_*7*_La_3_Zr_2_O_12_ solid electrolytes

**DOI:** 10.1038/s41598-020-79919-2

**Published:** 2021-01-11

**Authors:** Navaratnarajah Kuganathan, Michael J. D. Rushton, Robin W. Grimes, John A. Kilner, Evangelos I. Gkanas, Alexander Chroneos

**Affiliations:** 1 Department of Materials, Imperial College London, London, SW7 2AZ UK; 2 Faculty of Engineering, Environment and Computing, Coventry University, Priory Street, Coventry, CV1 5FB UK; 3 Nuclear Futures, Bangor University, Bangor, LL57 1UT Gwynedd UK; 4 International Institute for Carbon-Neutral Energy Research (I2CNER) Kyushu University, Fukuoka, 819-0395 Japan

**Keywords:** Batteries, Computational chemistry

## Abstract

Tetragonal garnet-type Li_*7*_La_3_Zr_2_O_12_ is an important candidate solid electrolyte for all-solid-state lithium ion batteries because of its high ionic conductivity and large electrochemical potential window. Here we employ atomistic simulation methods to show that the most favourable disorder process in Li_*7*_La_3_Zr_2_O_12_ involves loss of Li_2_O resulting in lithium and oxygen vacancies, which promote vacancy mediated self-diffusion. The activation energy for lithium migration (0.45 eV) is much lower than that for oxygen (1.65 eV). Furthermore, the oxygen migration activation energy reveals that the oxygen diffusion in this material can be facilitated at higher temperatures once oxygen vacancies form.

## Introduction

The quest for better capacity, cycle performance, safety and durability have led to the search for materials enabling completely solid-state lithium batteries^1–3^. A critical component in achieving this goal is the solid-state electrolyte at the heart of such batteries. The research community has considered numerous candidate materials including: anti-perovskite (Li_3_ClO and Li_7_P_3_S_11_)^4–6^, perovskites (La_2/3−x_Li_3x_TiO_3_)^7,8^, NASICON-type phosphates^9^, LiSICON^10^, thio-LiSICON^11,12^, LiBH_4_^13^, Li_9.54_Si_1.74_P_1.44_S_11.7_Cl_0.3_^14^, and the garnet family^15–20^.

A decade ago Muragan et al*.*^15^ introduced the garnet-type Li_*7*_La_3_Zr_2_O_12_ (LLZO) solid electrolyte with relatively high Li-ion conductivity, at room temperature, and in air. Lithium diffusion in both the tetragonal and cubic phases of LLZO and the impact of doping has been studied using experimental and theoretical methods^21–26^. The cubic phase of LLZO exhibits much higher Li-ion conductivity^18^ and doping (for example with Al, Ta, Ga) can stabilize the cubic phase to a lower temperature and enhance ionic conductivity^27–34^. Although there has been a focus on the cation sublattice and the improvement of Li-ion conductivity via doping, the impact of the oxygen sublattice is rarely considered. At high temperatures, which are important in fabricating garnet-type LLZO, there is loss of Li via formation of volatile Li compounds (e.g. Li_2_O) and this in turn leads to the formation of both Li and O vacancies^26,35^. Interestingly, in recent experimental work Kubicek et al*.*^26^ employed isotope exchange 3D imaging to measure oxygen diffusivity and infer the presence of oxygen vacancies in doped LLZO. These in turn impact phase stabilization and Li-ion diffusion^26^. Here, we investigate intrinsic defect processes and vacancy self-diffusion in tetragonal garnet-type Li_*7*_La_3_Zr_2_O_12_ solid electrolytes using two techniques. First, static atomistic simulation based on classical pair potentials are used to calculate intrinsic defect reaction energies and predict the most favourable dissorder process. This informs the choice of mediating defects that facilitate migration pathways for Li and O ions. Second, electronic structure calculations based on density functional theory (DFT) are employed to investigate the densities of states of the point defects (vacancy and interstitial) compared to defect-free LLZO.

## Results and discussion

### Calculation of the bulk Li_***7***_La_3_Zr_2_O_12_ structure

**Figure 1:**
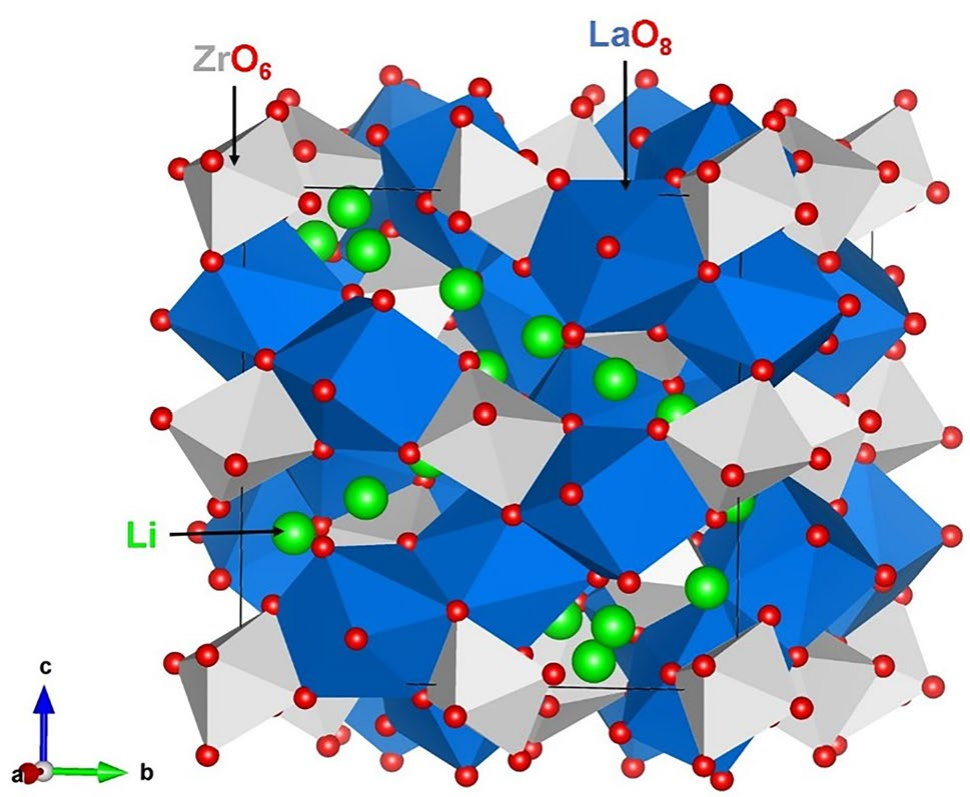
Crystal structure of tetragonal Li_7_La_3_Zr_2_O_12_ (space group I4_1_/acd). Li, La, Zr and O atoms are shown as green, blue, grey and red spheres, respectively.

The starting point for the present study is to use both computational approaches to reproduce the experimentally observed tetragonal crystal structure (space group I4_1_/acd) of Li_7_La_3_Zr_2_O_12_ (see Fig. 1) as reported by Logéat et al.^36^. This enables an assessment of the quality of the interatomic potential parameters used in the classical simulations, but also the pseudopotentials and basis set used for Li, La, Zr and O in the DFT simulations (see Table S1 in the Supplementary Information for the pair potentials parameters used and method section for the detailed descriptions of the methodologies). The calculated equilibrium lattice constants (see Table 1) obtained from both methods are in good agreement with experiment.

**Table 1:** Calculated and experimental lattice parameters of tetragonal Li_7_La_3_Zr_2_O_12_ (space group I4_1_/acd).

Parameter	Calculated		Experiment [^36^]	|∆| (%)	
	Classical	DFT		Classical	DFT
a = b (Å)	12.907	13.113	13.092	1.41	0.16
c (Å)	12.624	12.556	12.618	0.05	0.49
α = β = γ (°)	90.0	90.0	90.0	0.00	0.00

### Intrinsic defect processes

Greater insight into the intrinsic defect properties of electrolyte materials is crucial to fully understand their electrochemical behaviour and the rate of diffusion of their constituent ions. Using the classical pair potential simulations, a series of isolated point defect (vacancy and interstitial) energies were calculated, which were then combined to determine the formation energies for Frenkel, Schottky and antisite disorder processes in Li_7_La_3_Zr_2_O_12_. A 2 × 2 × 2 supercell containing 1536 atoms was used for the defect calculations. In the tetragonal LLZO crystal structure there are three non-equivalent Wyckoff positions that Li ions occupy (8a, 16f and 32g) and two non-equivalent positions on which La ions are present (8b and 16e). There is only one Wyckoff position for Zr (16c) and one for O (32g). Our calculations considered all non-equivalent sites for the formation of isolated vacancies. A number of interstitial positions were considered for all four ions. In both cases the lowest formation energies were taken into account to form the Schottky and Frenkel disorder reaction energies. The following equations, in Kröger–Vink notation^37^, represent the reactions involving these defects

1 $$ {\text{Li}}\;{\text{Frenkel:}}\quad {\text{Li}}_{{{\text{Li}}}}^{{\text{X}}} \to V_{{{\text{Li}}}}^{^{\prime}} + {\text{Li}}_{{\text{i}}}^{ \cdot } $$

2 $$ {\text{O}}\;{\text{Frenkel:}}\quad {\text{O}}_{{\text{O}}}^{{\text{X}}} \to V_{{\text{O}}}^{ \cdot \cdot } + {\text{O}}_{{\text{i}}}^{\prime \prime } $$

3 $$ {\text{La}}\;{\text{Frenkel:}}\quad {\text{La}}_{{{\text{La}}}}^{{\text{X}}} \to V_{{{\text{La}}}}^{\prime \prime \prime } + {\text{La}}_{{\text{i}}}^{ \cdot \cdot \cdot } $$

4 $$ {\text{Zr}}\;{\text{Frenkel:}}\quad {\text{Zr}}_{{{\text{Zr}}}}^{{\text{X}}} \to V_{{{\text{Zr}}}}^{\prime \prime \prime \prime } + {\text{Zr}}_{{\text{i}}}^{ \cdot \cdot \cdot \cdot } $$

5 $$ {\text{Schottky:}}\quad 7\;{\text{Li}}_{{\text{Li }}}^{{\text{X}}} + 3\;{\text{La}}_{{{\text{La}}}}^{{\text{X }}} + 2\;{\text{Zr}}_{{{\text{Zr}}}}^{{\text{X}}} + 12\;{\text{O}}_{{\text{O}}}^{{\text{X}}} \to 7\;V_{{{\text{Li}}}}^{\prime } + 3\;V_{{{\text{La}}}}^{\prime \prime \prime } + 2\;V_{{{\text{Zr}}}}^{\prime \prime \prime \prime } + 12\;V_{{\text{O}}}^{ \cdot \cdot } + {\text{Li}}_{7} {\text{La}}_{3} {\text{Zr}}_{2} {\text{O}}_{12} $$

6 $$ {\text{Li}}_{2} {\text{O}}\;{\text{Schottky:}}\quad 2\;{\text{Li}}_{{{\text{Li}}}}^{{\text{X}}} + {\text{O}}_{{\text{O}}}^{{\text{X }}} \to {2}V_{{{\text{Li}}}}^{\prime } + V_{{\text{O}}}^{ \cdot \cdot } + {\text{Li}}_{2} {\text{O}} $$

7 $$ {\text{La}}_{2} {\text{O}}_{3} \;{\text{Schottky:}}\quad 2\;{\text{La}}_{{\text{La }}}^{{\text{X}}} + 3{\text{O}}_{{\text{O}}}^{{\text{X }}} \to 2V_{{{\text{La}}}}^{\prime \prime \prime } { } + { }3{ }V_{{\text{O}}}^{ \cdot \cdot } + {\text{La}}_{2} {\text{O}}_{3} $$

8 $$ {\text{ZrO}}_{2} \;{\text{Schottky:}}\quad {\text{Zr}}_{{{\text{Zr}}}}^{{\text{X}}} + 2\;{\text{O}}_{{\text{O}}}^{{\text{X }}} \to V_{{{\text{Zr}}}}^{\prime \prime \prime \prime } + 2\;V_{{\text{O}}}^{ \cdot \cdot } + {\text{ZrO}}_{2} $$

9 $$ {\text{Li/La}}\;{\text{antisite }}\left( {{\text{isolated}}} \right){:}\quad {\text{Li}}_{{{\text{Li}}}}^{{\text{X}}} + {\text{La}}_{{{\text{La}}}}^{{\text{X }}} \to {\text{Li}}_{{{\text{La}}}}^{\prime \prime } + {\text{La}}_{{{\text{Li}}}}^{ \cdot \cdot } $$

10 $$ {\text{Li/La}}\;{\text{antisite }}\left( {{\text{pair}}} \right){:}\quad {\text{Li}}_{{{\text{Li}}}}^{{\text{X}}} + {\text{La}}_{{{\text{La}}}}^{{\text{X}}} \to \left\{ {{\text{Li}}_{{{\text{La}}}}^{\prime \prime } {\text{:La}}_{{{\text{Li}}}}^{ \cdot \cdot } } \right\}^{{\text{X}}} $$

11 $$ {\text{Li/Zr}}\;{\text{antisite }}\left( {{\text{isolated}}} \right){:}\quad {\text{Li}}_{{{\text{Li}}}}^{{\text{X}}} + {\text{Zr}}_{{{\text{Zr}}}}^{{\text{X}}} \to {\text{Li}}_{{{\text{Zr}}}}^{\prime \prime \prime } + {\text{Zr}}_{{{\text{Li}}}}^{ \cdot \cdot \cdot } $$

12 $$ {\text{Li/Zr}}\;{\text{antisite }}\left( {{\text{pair}}} \right){:}\quad {\text{Li}}_{{{\text{Li}}}}^{{\text{X}}} + {\text{Zr}}_{{{\text{Zr}}}}^{{\text{X}}} \to \left\{ {{\text{Li}}_{{{\text{Zr}}}}^{\prime \prime \prime } {\text{:Zr}}_{{{\text{Li}}}}^{ \cdot \cdot \cdot } } \right\}^{{\text{X}}} $$

**Figure 2:**
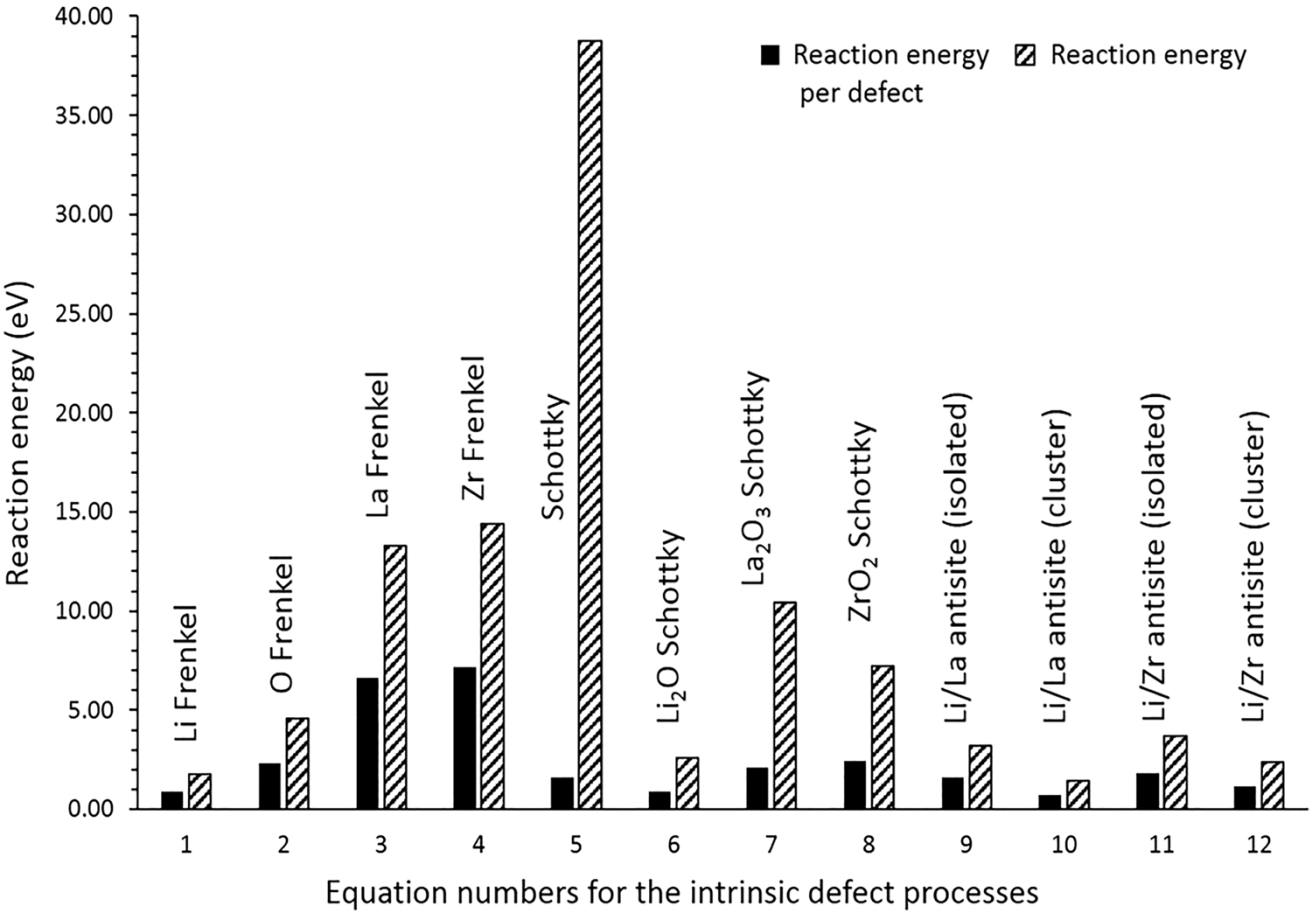
Energetics of intrinsic defect process in tetragonal Li_7_La_3_Zr_2_O_12_.

Reaction energies (normalised per defect) for these intrinsic defect processes, calculated using the classical pair potential approach, are shown in Fig. 2. The formation of all Frenkel and Schottky defects is unfavourable, suggesting that the formation of vacancies (especially La and Zr but as we will see not Li) and interstitial defects is unfavourable on energetic grounds. Hence, such intrinsic disorder is unlikely to be present in high concentrations in undoped Li_*7*_La_3_Zr_2_O_12._

Of the Frenkel processes, Li Frenkel is of dramatically lower energy than the other cation Frenkel reactions and less than half that for O Frenkel. The enthalpy to form Li_2_O (i.e. the removal of Li_2_O through a Schottky-like, or partial Schottky process) is also a relatively low energy process compared to other Schottky reactions in LLZO. DFT simulation performed by Moradabadi et al.^38^ on Al-doped cubic LLZO shows that Li_2_O Schottky-like defect comprising of a cluster of 2 Li^+^ and O^2−^ vacancies is the second most favourable defect process under poor O condition though the most favourable defect type is ZrLi_2_O-Schottky-like defect under poor Zr condition. Simulation studies indicates that the loss of Li_2_O seems to be a common feature in pristine and doped-LLZO. Furthermore, it was shown that the formation of isolated point defects ($V_{{{\text{Li}}}}^{\prime }$ and ${\text{Li}}_{{\text{i}}}^{ \cdot }$) is unfavourable and their co-existence ($V_{{{\text{Li}}}}^{\prime }$ + ${\text{Li}}_{{\text{i}}}^{ \cdot }$ or Li Frenkel) is energetically favourable. A key feature of Li_*7*_La_3_Zr_2_O_12_ is that, in principle, extraction of more than one lithium is possible in contrast to olivine phosphates^39^ in which one lithium at most can be extracted per formula unit though the exact number of Li that can be extracted without collapsing the crystal structure is not known experimentally. When more than one Li^+^ is extracted from LLZO, the material will keep its neutrality by losing O^2−^ ions according the reaction Eq. (6). An experimental study carried out by Kubicek et al.^26^ shows that there is a substantial level of oxygen diffusion in LLZO with an oxygen nonstoichiometry of ~ 5 × 10^−3^ per unit cell as a first rough estimate on the single crystal.

The O Frenkel in the current study is slightly lower in energy than the values calculated for the olivine phosphate^40,41^ and for orthosilicate^42^ based lithium ion battery materials. The lower formation enthalpy of Li_2_O in this material may be due to the high Li content in this material. Also the lower Li Frenkel reaction energy indicates that the loss of Li_2_O at high temperatures can be facilitated via the formation of a compensating O vacanc**y**. Johnson et al.^43^ have observed a change in coordination of Fe^3+^ ion in the delithiated samples of Li_5_FeO_4_ suggesting that lithium extraction is predominantly assisted by the release of O with the net loss of Li_2_O leaving a Fe_2_O_3_ rich residual product.

We also considered the Li/M (M = La and Zr) “anti-site” isolated and pair defects. In the isolated cases, the defect energy for the two dopant substitutions were calculated separately then used in Eqs. (9) and (11) to give the energy of the overall defect process (meaning that the effects of defect association were not included). In the case of the clustered “anti-site” pair (Eqs. 10 and 12) a Li^+^ ion (radius 0.76 Å) exchanges with a La^3+^ ion (radius 1.16 Å) or Zr^4+^ (radius 0.72 Å). Substitution was considered on all non-equivalent sites of the metals and the lowest energy was taken to calculate isolated (association) energies. A number of defect cluster configurations in which Li ions neighbouring La ions or Zr ions were considered and the lowest defect energy was for the smallest cation–cation separation and is reported as the cluster defect energy. As interactions between isolated point defects can lead to the formation of clusters, the cluster binding energies were calculated using the following equation

13 $${E}_{bind}={E}_{cluster}-\sum {E}_{isolated}$$

The energies in Fig. 2 reveal that the “anti-site” cluster pair defect is a lower energy process compared to its isolated form. This means that a small percentage of Li^+^ ions can exchange with La^3+^ or Zr^4+^. The exact concentration will depend on the temperature and synthesis procedure. The cluster binding energies for the defect clusters $\left\{ {Li_{La}^{\prime \prime } {:}La_{Li}^{ \cdot \cdot } } \right\}^{X}$ and $\left\{ {Li_{Zr}^{\prime \prime \prime } {:}Zr_{Li}^{ \cdot \cdot \cdot } } \right\}^{X}$ were calculated to be − 0.88 eV and − 0.66 eV per defect, respectively. Both Li/La and Li/Zr antisite pair clusters have negative binding energies meaning that they are more stable than the isolated defects. Cation exchange effects have also been predicted in polyanionic based Li ion battery materials^40–42,44^. Further, the presence of such defects has been confirmed by Chung et al*.*^45^ and Politaev et al*.*^46^ in their experimental studies of olivine-type LiFePO_4_ and in monoclinic Li_2_MnSiO_4_, respectively.

### Self-diffusion

**Figure 3:**
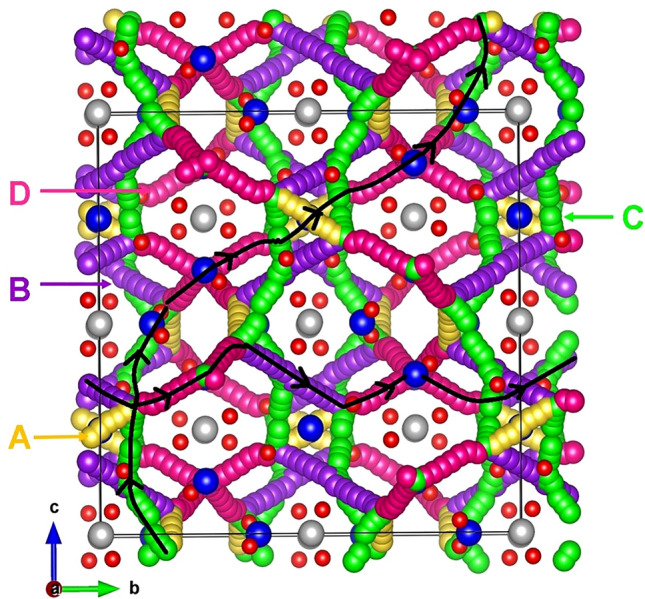
Possible long range lithium vacancy migration paths. Grey, blue and red colours correspond to Zr, La and O atoms, respectively. Four different Li local hops (A–D) are shown in different colours.

**Figure 4:**
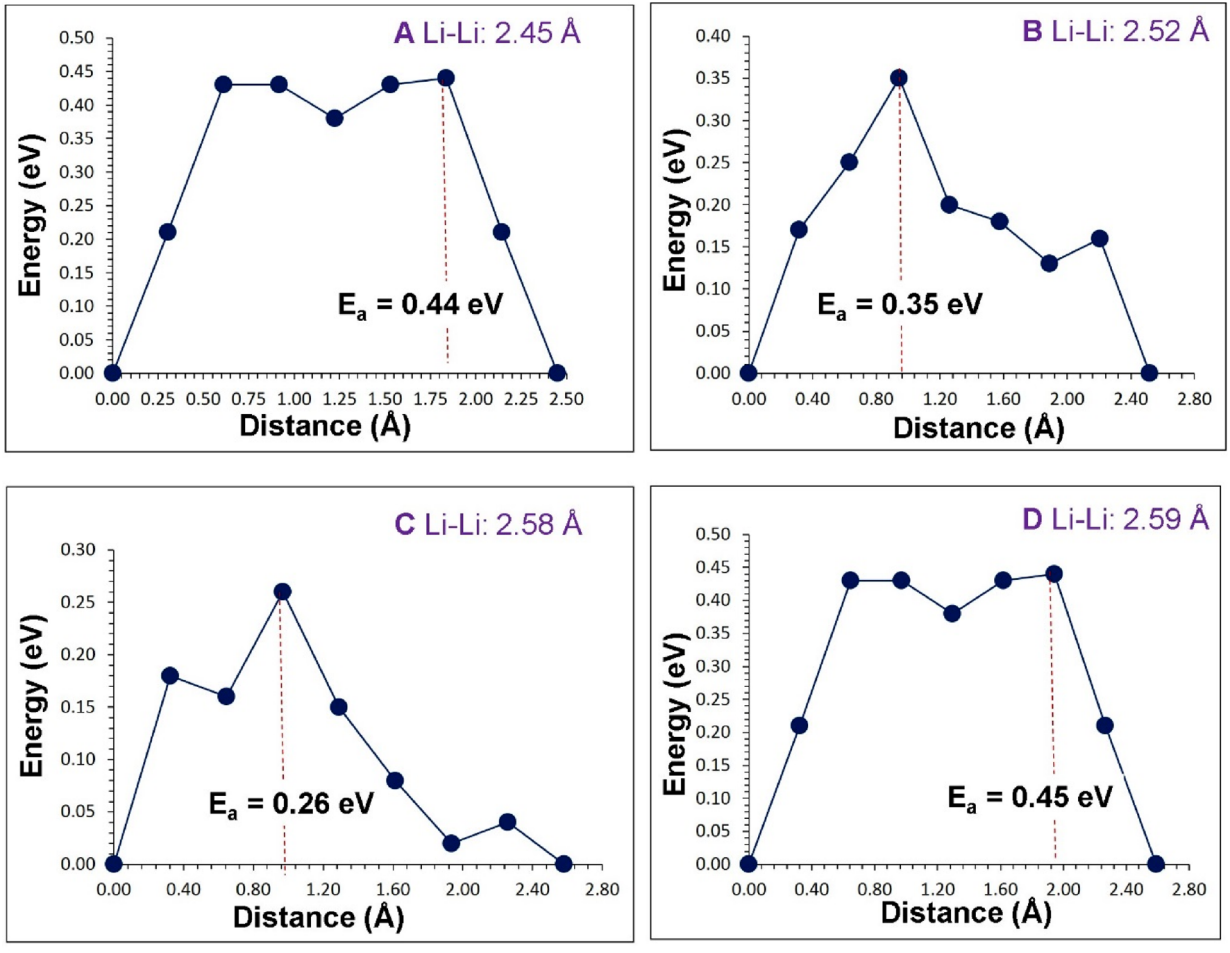
Four different energy profiles for Li vacancy hops (as shown in Fig. 3) in Li_7_La_3_Zr_2_O_12_. E_a_ corresponds to the activation energy for Li ion migration.

**Figure 5:**
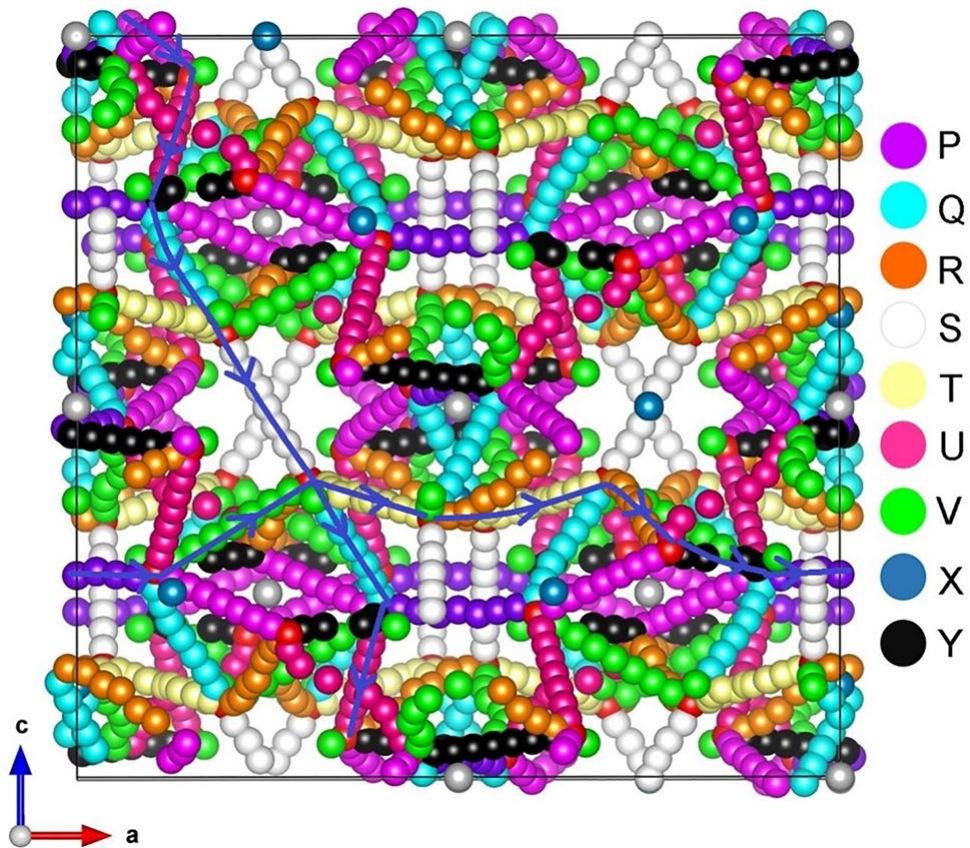
Possible long range oxygen vacancy migration paths. Nine different O local hops (P–Y) are shown in different colours.

**Figure 6:**
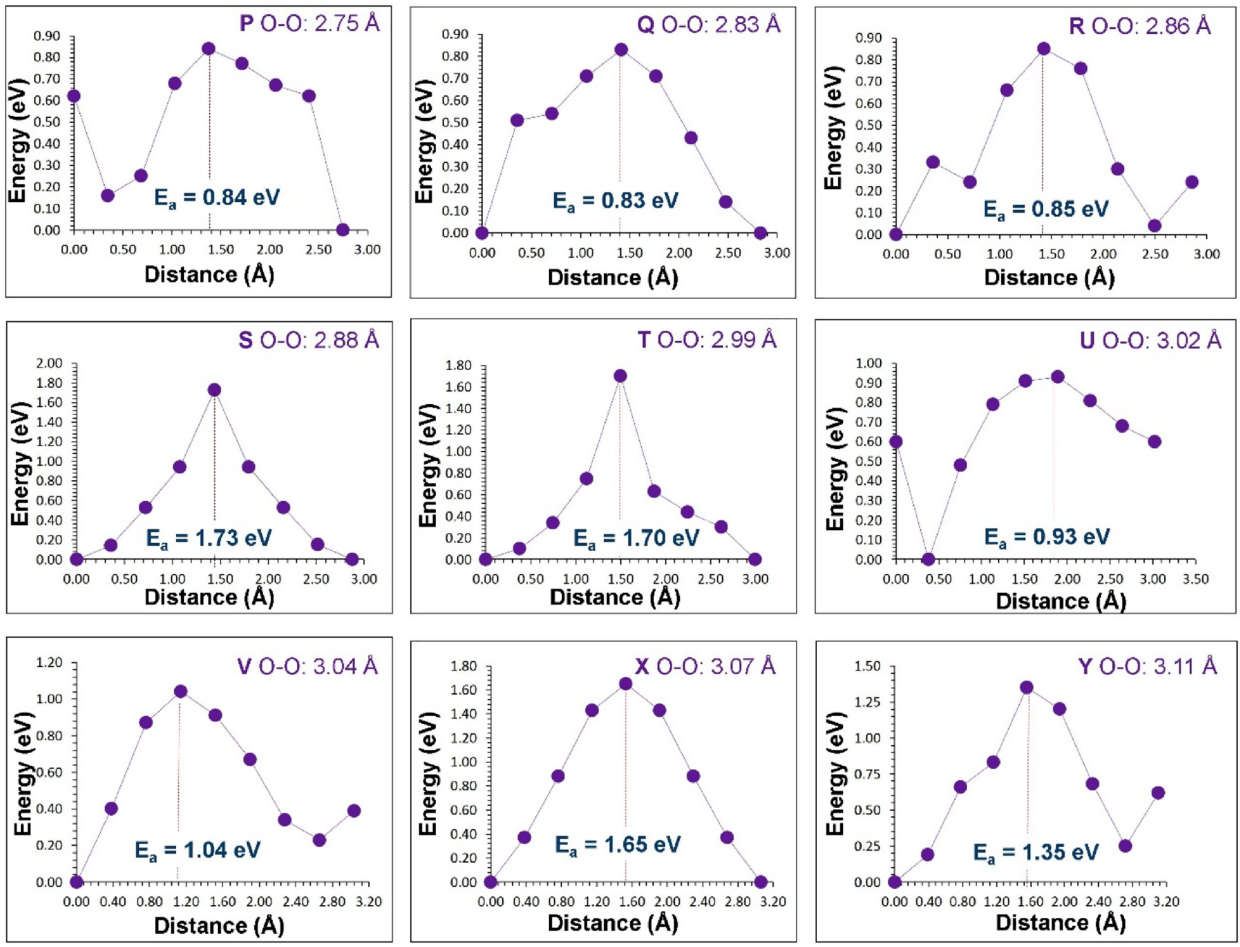
Nine different energy profiles for O vacancy hops (as shown in Fig. 5) in Li_7_La_3_Zr_2_O_12_.

The previous section established that at high temperatures there is a tendency for Li_*7*_La_3_Zr_2_O_12_ to lose lithium in the form of Li_2_O with the concomitant formation of lithium and oxygen vacancies. These can become vehicles for lithium and oxygen self-diffusion, respectively via the vacancy mechanism. Current classical simulation allowed us to examine possible Li and O vacancy migration paths and calculate corresponding activation energies. Figures 3 and 4 represent the potential migration pathways including all possible jumps between adjacent lithium sites and energy profile diagrams for the corresponding Li hops (A–D), respectively. Figures 5 and 6 show the local O hops (P–Y) and corresponding energy profile diagrams, respectively. The position of highest potential energy (i.e. the “saddle point” configuration) along the migration path determines the migration activation energy (E_a_). The calculated Li and O activation for individual jumps are reported in Tables 2 and 3, respectively.

**Table 2:** Calculated Li–Li separations and activation energies for lithium ion migration. For migration paths refer to Fig. 3.

Migration path	Li–Li separation (Å)	Activation energy (E_a_) (eV)
A	2.45	0.44
B	2.52	0.35
C	2.58	0.26
D	2.59	0.45

**Table 3:** Calculated O–O separations and activation energies for the oxygen ion migration.

Migration path	O–O separation (Å)	Activation energy (E_a_) (eV)
P	2.75	0.84
Q	2.83	0.83
R	2.86	0.85
S	2.88	1.73
T	2.99	1.70
U	3.02	0.93
V	3.04	1.04
X	3.07	1.65
Y	3.11	1.35

For migration paths refer to Fig. 5.

For the Li migration, we identified four main local paths (hops) between adjacent Li sites (see Fig. 3). The lowest migration barrier is 0.26 eV (path C) but this path alone cannot facilitate transport of Li across the unit cell. Individual migration paths (A–D) were then connected to construct long range Li diffusion channels (i.e. contiguous across the whole unit cell). Two lowest energy 3D transport of Li ion pathways (see Fig. 3) were identified. In both cases the overall activation energy of Li ion migration is 0.45 eV. The calculated activation energy is in good agreement with the experimental activation energy of 0.54 eV calculated in tetragonal Li_*7*_La_3_Zr_2_O_12_ by Awaka et al.^47^ in the temperature range of 300–560 K and a DFT calculated activation energy of 0.44 eV by Meier et al*.*^48^. Consistently with previous experimental and theoretical work the activation energy calculated here for the tetragonal phase is higher than the cubic phase (0.29 eV)^34^.

Next we considered all possible O vacancy hops between adjacent O ions in the lattice. Nine possible migration hops [(P–Y) in Fig. 5] led to two long range low energy paths. The migration energies for hops ranged between 0.83 and 1.73 eV. For the two long range O ion migration pathways (X → V → T → T → R → Y → X) and (P → U → Q → S → Q → U), as shown in the Fig. 5, the activation energies were 1.65 eV and 1.73 eV, respectively. It is important to note that the activation energies for Li local hops (A–D) are lower than for the O local hops (P–Y) as are the energies for the overall pathways. This suggests that Li transport will be considerably more favourable than that of O in this material.

### Densities of states

**Figure 7:**
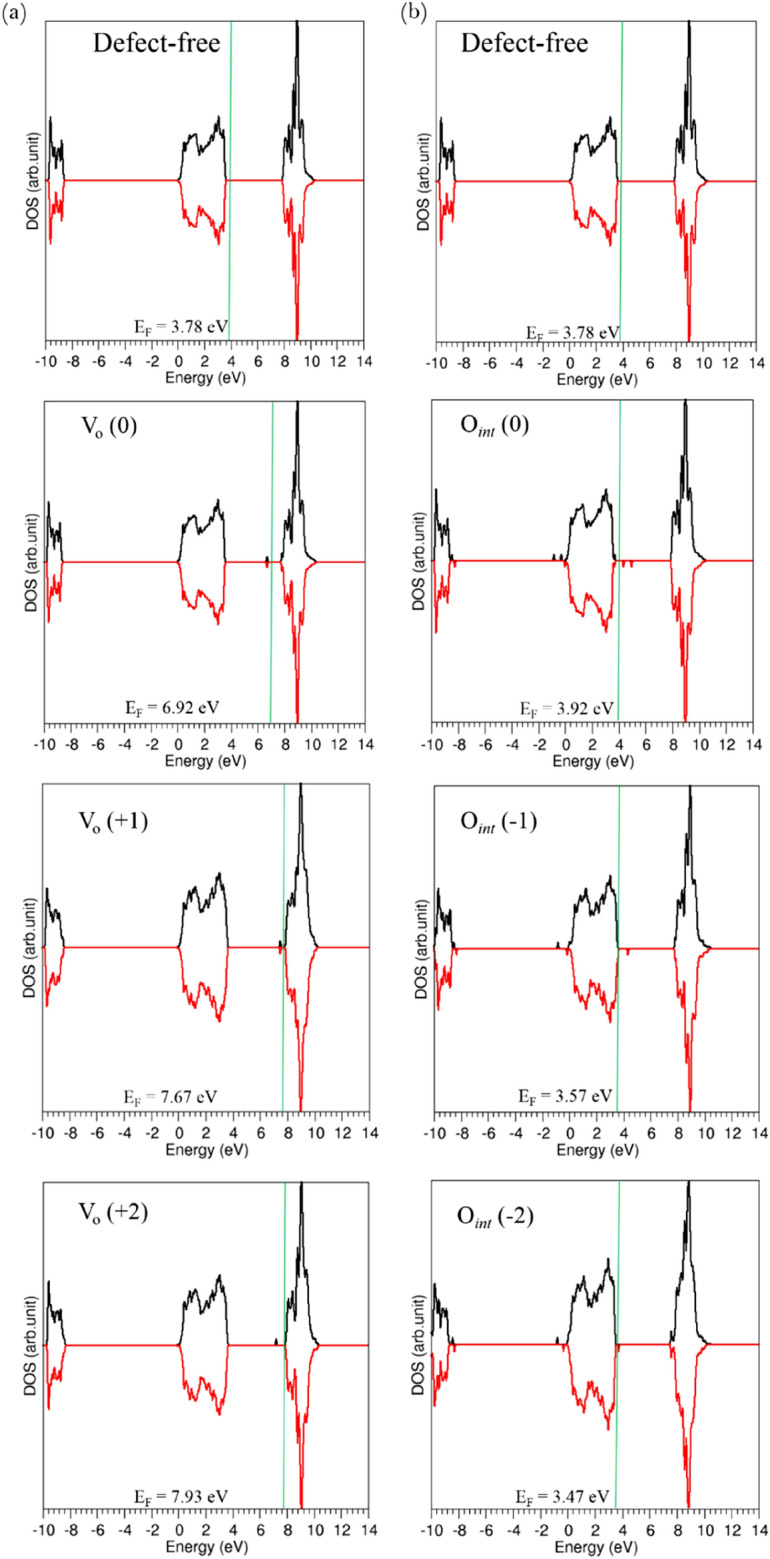
DOSs for tetragonal Li_7_La_3_Zr_2_O_12_ with (**a**) oxygen vacancies, and (**b**) oxygen interstitial defects in different charge states. The Fermi energies (E_F_) are given in each cases and indicated by green solid lines.

The DFT based simulations predict that Li_7_La_3_Zr_2_O_12_ is a wide-gap semiconductor with the band gap of 4.50 eV (refer to Fig. 7). Thompson et al.^49^ have calculated the band gap and density of states of Li_7_La_3_Zr_2_O_12_ using PBE, HSE06 and G0W0 levels of theory and their calculated band gaps were 4.33 eV, 5.79 eV and 6.42 eV respectively, agreeing well with the value reported here. In order to analyse the influence of the defects (vacancies and interstitials) on the electronic structure of Li_7_La_3_Zr_2_O_12_, the electronic densities of states (DOSs) for both defect-free and defective Li_7_La_3_Zr_2_O_12_ were determined, as shown in Fig. 7. The formation of vacancies or interstitial ions does not significantly change the DOS of Li_7_La_3_Zr_2_O_12_ but introduces new defect energy levels in the band gap of defective material. The neutral oxygen vacancy defect shifts the Fermi energy significantly (see Fig. 7a) from the valence band maximum in defect free Li_7_La_3_Zr_2_O_12_ to above the defect energy level of the V_O_ (0) defect. A further shift in the Fermi energy is observed for oxygen vacancies with + 1 and + 2 charges and the defect energy levels are still present above the top of the valence band maximum but closer to the conduction band minimum. For the system with a neutral interstitial oxygen defect, the Fermi energy shifts slightly from the valence band maximum. In the case of oxygen vacancy defects with − 1 and − 2 charges, the Fermi energy shifts back towards the valence band maximum and these values are slightly below the Fermi energy level observed for pure Li_7_La_3_Zr_2_O_12_.

### Summary

Intrinsic defect processes and vacancy self-diffusion in garnet-type Li_*7*_La_3_Zr_2_O_12_ solid electrolytes have been predicted using atomic scale computer simulation. The removal of a Li_2_O formula unit via a Schottky-like process exhibited an especially low enthalpy: 0.87 eV/defect. This implies that high temperatures, such as those encountered in materials processing, will lead to Li_2_O volatilisation and hence the formation of both Li and oxygen vacancies (i.e. a Li substoichiometric compostion). This would only be mitigated in cases where there is an excess Li_2_O to maintain a high vapour pressure processing atmosphere. The presence of oxygen vacancies has already been inferred from oxygen diffusion experiments. The vacancy migration activation energy for oxygen self-diffusion, was predicted to be 1.65 eV. Thus lithium transport is favoured over oxygen transport in a Li_2_O deficient material.

## Methods

### The classical method

The general utility lattice program (GULP)^50^ was used to carry out a detailed survey of the relative energetics for the formation of intrinsic defects and to identify the possible pathways for lithium and oxygen ion conduction. This atomic scale simulation code is based on the Born model of solids. All systems are treated as crystalline solids, with interactions between ions consisting of a long-range Coulombic term and a short-range component representing electron–electron repulsion and van der Waals interactions. The short-range interactions used the Buckingham potential reported by Jalem et al.^17^ Simulation boxes and the corresponding atom positions were relaxed using the Broyden–Fletcher–Goldfarb–Shanno (BFGS) algorithm^51^. The Mott–Littleton method^52^ was used to investigate the lattice relaxation about point defects or migrating ions. It divides the crystal lattice into two concentric spherical regions, where the ions within the inner spherical region immediately surrounding the defect are relaxed explicitly. The remainder of the crystal, where the defect forces are relatively weak, is treated by more approximate quasi-continuum methods. In this way local relaxation is modelled effectively and the crystal is not considered as simply a rigid lattice. The defect calculations used region sizes of 10 Å and 20 Å for inner and outer regions, respectively.

Li and O migration calculations were performed within the Mott–Littleton framework creating two adjacent Li (or O) vacancy sites and systematically placing an Li (or O) ion at regular intervals along the diagonal connecting them. Seven interstitial positions were considered in all cases and the interstitial ion was fixed while all other ions were free to relax. The difference in energy between the saddle point position and the system in its initial state was calculated and reported as the activation energy. The current methodology to calculate migration pathways has been discussed in detail in previous studies and applied to a number of ionic oxide materials^53–56^.

### The quantum mechanical method

In order to calculate the electronic DOSs for pure and defective (O vacancy and interstitial) LLZO, ab initio total energy calculations were employed, based on the spin polarised mode of DFT as implemented in the Vienna Ab initio simulation package (VASP)^57,58^. The standard projected augmented wave (PAW) potentials^59^ and a plane-wave basis set with a cut off value of 500 eV were used in all cases. The exchange–correlation term was modelled using the generalised gradient approximation (GGA), parameterised by Perdew, Burke and Ernzerhof (PBE)^60^. The valence electronic configurations for Li, La, Zr and O were 1s^2^ 2s^1^, 5s^2^ 5p^6^ 5d^1^ 6s^2^, 4s^2^ 4p^6^ 4d^2^ 5s^2^ and 2s^2^ 2p^4^, respectively. A primitive unit cell containing 192 atoms was used to model oxygen vacancy and interstitial defects. Geometery optimisations were performed using a 2 × 2 × 2 Monkhorst–Pack^61^ *k* point mesh (which yields 8 k points) and a 4 × 4 × 4 k point mesh (which yields 36 k points) was used to calculate the DOS. Structural optimisations were performed using a conjugate gradient algorithm^62^ and the forces on the atoms were obtained from the Hellman–Feynmann theorem including Pulay corrections. In all optimized structures, forces on the atoms were smaller than 0.001 eV/Å and the stress tensor was less than 0.002 GPa. In this work, dispersion has been included by using the pair-wise force field as implemented by Grimme et al.^63^ in the VASP package.

## Supplementary Information

Supplementary Information.
