# Supplementary material for: Self-diffusion in garnet-type Li7La3Zr2O12 solid electrolytes
Source: Sci Rep. 2021 Jan 11;11:451. doi: 10.1038/s41598-020-79919-2 (PMC7801511; doi:10.1038/s41598-020-79919-2)
Supplement: Supplementary file 1 — Supplementary Information. [file 41598_2020_79919_MOESM1_ESM.docx]

Supplementary Information

**Self-diffusion in garnet-type Li*_7_*La_3_Zr_2_O_12_ solid electrolytes**

# Navaratnarajah Kuganathan,^1,2*^ Michael J. D. Rushton,^3^ Robin W. Grimes,^1^ John A. Kilner,^1,4^ Evangelos I. Gkanas,^2^ Alexander Chroneos,^1,2*^

^1^*Department of Materials, Imperial College London, London SW7 2AZ, United Kingdom*

^2^*Faculty of Engineering, Environment and Computing, Coventry University, Priory Street, Coventry CV1 5FB, United Kingdom*

^3^*Nuclear Futures, Bangor University, Bangor, Gwynedd, LL57 1UT, United Kingdom*

^4^*International Institute for Carbon-Neutral Energy Research (I2CNER) Kyushu University, Fukuoka 819-0395, Japan*

*Correspondence to [n.kuganathan@imperial.ac.uk](mailto:n.kuganathan@imperial.ac.uk) or [alexander.chroneos@imperial.ac.uk](mailto:alexander.chroneos@imperial.ac.uk)

**Table S1:** Reported interatomic potential parameters^1^ used in this study to model short range interactions using the Buckingham’s two-body potential [Φ*_ij_* (*r_ij_*) = *A_ij_* exp (− *r_ij_*/*ρ_ij_*) − *C_ij_ / r_ij_*^6^].

| Interaction | A_ij_ (eV) | ρ_ij_ (Å) | C_ij_ (eV·Å^6^) |
| --- | --- | --- | --- |
| Li^0.7+^ - O^1.4–^ | 876.86 | 0.2433 | 0.00 |
| La^2.1+^ - O^1.4–^ | 14509.63 | 0.2438 | 30.83 |
| Zr^2.8+^ - O^1.4–^ | 2153.80 | 0.2908 | 0.00 |
| O^1.4−^ - O^1.4–^ | 4869.99 | 0.2402 | 27.22 |

**Table S2.** Calculated and experimental structural parameters for cubic (Fm$\overline{3}m$) Li_2_O

| Parameter | Calc (classical) | Expt^2^ | \|∆\| (%) |
| --- | --- | --- | --- |
| a = b = c (Å) | 4.503 | 4.566 | 1.40 |
| α = β = γ (°) | 90.0 | 90.0 | 0.00 |

**Table S3.** Calculated and experimental structural parameters for cubic (Ia$\overline{3}$) La_2_O_3_

| Parameter | Calc (classical) | Expt^3^ | \|∆\| (%) |
| --- | --- | --- | --- |
| a = b = c (Å) | 11.232 | 11.387 | 1.36 |
| α = β = γ (°) | 90.0 | 90.0 | 0.00 |

**Table S4.** Calculated and experimental structural parameters for tetragonal (P4_2_/nmc) ZrO_2_

| Parameter | Calc (classical) | Expt^4^ | \|∆\| (%) |
| --- | --- | --- | --- |
| a = b (Å) | 3.649 | 3.596 | 1.47 |
| c (Å) | 5.160 | 5.184 | 0.46 |
| α = β = γ (°) | 90.0 | 90.0 | 0.00 |

**References**

1. Jalem, R. *et al.* Effects of gallium doping in garnet-type Li_7_La_3_Zr_2_O_12_ solid electrolytes.

*Chem. Mater.* **27**, 2821-2831 (2015).

2. Hull, S. *et al*. The elastic properties of lithium oxide and their variation with temperature.

*J. Nucl.Mater*. **160**, 125-134 (1988).

3. Rustad, J. R. Density functional calculations of the enthalpies of formation of rare-earth

orthophosphates. *Am. Mineral*. **97**, 791-799 (2012).

4. Bondars, B. Y. *et al*. Powder diffraction investigations of plasma sprayed zirconia.*Mater.*

*Sci*. **30**, 1621-1625 (1995).
